# Correlation of Early Vascular Aging Ambulatory Score with Kidney Damage in a Hypertensive Population: A Pilot Study

**DOI:** 10.3390/life16030504

**Published:** 2026-03-19

**Authors:** Georgios Samprokatsidis, Christina Antza, Ioannis Partheniadis, Smaro Palaska, Panagiota Anyfanti, Vasilios Kotsis

**Affiliations:** 1 3rd Department of Internal Medicine, Medical School, Aristotle University of Thessaloniki, 541 24 Thessaloniki, Greece; georgesabro@gmail.com (G.S.); kris-antza@hotmail.com (C.A.); smaropal@gmail.com (S.P.); panyfan@hotmail.com (P.A.); 2 Department of Pharmaceutical Technology, School of Pharmacy, Faculty of Health Sciences, Aristotle University of Thessaloniki, 541 24 Thessaloniki, Greece; ioanpart@pharm.auth.gr

**Keywords:** early vascular aging, hypertension, chronic kidney disease, albuminuria, ambulatory blood pressure monitoring

## Abstract

Background: Early vascular aging (EVA) reflects accelerated arterial stiffening and is closely linked to cardiovascular and renal target organ damage. The Early Vascular Aging Ambulatory score (EVAAs) estimates EVA using ambulatory blood pressure monitoring (ABPM) and routinely available clinical parameters. We aim to investigate the association between EVAAs-defined early vascular aging and markers of kidney involvement—particularly albumin-to-creatinine ratio (ACR)—in a hypertensive population. Methods: Fifty treated hypertensive adults undergoing 24 h ABPM were enrolled. All participants underwent laboratory evaluation, including serum electrolytes and 24 h urine collection for albumin, creatinine, sodium, and potassium. EVAAs was calculated using ABPM-derived parameters and established cardiovascular risk factors. Results: EVAAs was positively correlated with ACR (*r* = 0.276, *p* = 0.049). In addition, inverse correlations were observed between EVAAs and serum potassium (*r* = −0.290, *p* = 0.038) and serum sodium (*r* = −0.284, *p* = 0.046). Participants with moderately increased albuminuria tended to exhibit higher EVAAs values, although this difference did not reach statistical significance. Conclusions: EVAAs is associated with early markers of renal involvement in hypertensive patients, supporting its potential role as a non-invasive indicator of subclinical target organ damage. Larger studies are warranted to confirm these findings and to further validate EVAAs as a clinically useful marker of EVA.

## 1. Introduction

Hypertension remains the most prevalent cardiovascular disorder worldwide and a leading contributor to cardiovascular morbidity and mortality. According to the World Health Organization (WHO), more than 1.2 billion adults are affected globally, with hypertension representing a major driver of target organ damage involving the heart, brain, vasculature, and kidneys [1]. Consequently, contemporary hypertension management emphasizes not only blood pressure (BP) control but also early detection of subclinical organ damage [2].

Arterial stiffness is a core pathophysiological component of vascular aging and a recognized marker of cardiovascular risk [3]. Increased arterial stiffness contributes to systolic hypertension, pulse pressure widening, and microvascular damage, promoting injury to highly perfused organs such as the kidneys [3]. Conversely, impaired renal function may exacerbate arterial stiffness through disturbances in volume regulation, mineral metabolism, inflammation, and endothelial dysfunction, highlighting a bidirectional relationship between vascular and renal damage [4].

Pulse wave velocity (PWV) is regarded as the gold-standard measure of arterial stiffness; however, its routine clinical use is limited by technical complexity, equipment requirements, and operator dependence [2,5]. Alternative indices, including the augmentation index and ambulatory arterial stiffness index, have been proposed, yet each has inherent limitations [6]. In this context, composite scoring systems integrating ambulatory BP data with clinical and laboratory variables have emerged as practical tools for estimating vascular aging [7].

The Early Vascular Aging Ambulatory score (EVAAs), introduced in 2018, combines 24 h systolic and diastolic BP, heart rate, age, sex, body mass index (BMI), diabetes status, and estimated glomerular filtration rate (eGFR) to estimate the probability of early vascular aging (EVA) [7]. EVAAs has demonstrated acceptable diagnostic accuracy and has been shown to approximate PWV-based vascular aging assessments [8]. Furthermore, it has exhibited prognostic value in specific clinical settings, such as ischemic stroke [9].

Despite growing interest in EVAAs, its association with renal target organ damage—particularly albuminuria, a sensitive marker of endothelial and microvascular dysfunction, but also chronic kidney disease (CKD)—has not been adequately studied. Albuminuria, even within the high normal range, has been linked to arterial stiffness and vascular aging in both hypertensive and normotensive populations [10,11].

Therefore, the aim of the present study was to explore the relationship between EVAAs and markers of renal involvement, with particular emphasis on albumin-to-creatinine ratio (ACR), in a population of treated hypertensive patients. The present study was designed as an exploratory pilot investigation focusing specifically on a clinically characterized population of treated hypertensive patients, with the aim of examining intra-group associations rather than performing case–control comparisons. This approach was intended to generate hypothesis-forming data to inform future larger and controlled studies.

## 2. Materials and Methods

### 2.1. Study Population

Subjects aged > 18 years old were selected as the study population. The patients had been admitted to the Hypertension 24 h Ambulatory Blood Pressure Monitoring (ABPM) Center of Excellence of the 3rd Internal Medicine Department of Aristotle University in Thessaloniki, Greece. The research protocol of the study was approved by both the institutional board and the academic board of the Aristotle University of Thessaloniki. The methodology followed during the study was in line with the declaration of Helsinki and all subjects who volunteered for the study gave their informed consent in order to participate.

**Figure 1:**
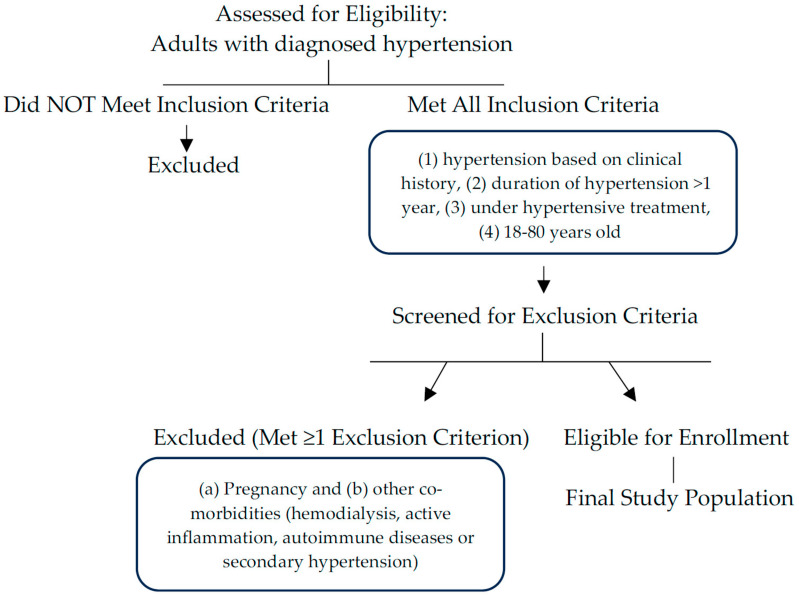
Study Selection Process: Inclusion and Exclusion Criteria.

The population of the study consisted of all subjects who were admitted to the hypertension center for elevated BP between 1 November 2023 and 31 January 2024. Participants were selected, regardless of gender and/or sexual orientation. Additionally, the term “sex” refers only to the biological factors of each subject. The inclusion criteria were: (1) hypertension based on clinical history; (2) duration of hypertension >1 year; (3) population under hypertensive treatment; (4) ages between 18 and 80 years old. The exclusion criteria were: (a) pregnancy; (b) other co-morbidities such as known CKD, hemodialysis, active inflammation, autoimmune diseases or secondary causes of hypertension (renovascular hypertension, etc.), as shown in Figure 1. Laboratory exams, weight, height, BMI, waist and hip circumference and a brief history regarding certain cardiovascular parameters were recorded. eGFR estimation, 24 h urine specimen measurements (24 h creatinine and albumin levels), and estimation of the albumin-to-creatinine ratio (ACR) were evaluated in all participants. Kidney function was evaluated using eGFR estimation, calculated by the “modification of diet in renal disease” (MDRD) formula [12,13].

### 2.2. Methods and Measurements

(i) BP (Office and 24 h)

All subjects included in the study had office BP measurements, which were performed 3 times consecutively with about 1–2 min between measurements with the use of a licensed BP sphygmomanometer at an appropriate cuff size for each subject, in a seated position. All measurements were performed according to hypertension guidelines [2,14].

ABPM was initiated during the same visit. Using a Spacelabs 90217 ambulatory BP monitor (Spacelabs, Redmond, WA, USA), 24 h ABPM was performed. The appropriate cuff size was used on the non-dominant arm of each subject. Additionally, 3 BP measurements were carried out to confirm the absence of variations in BP of >5 mmHg. Subjects were given instructions for the duration of the procedure as well as proper guidance for the functioning of the monitor to ensure proper acquirement of BP measurement with as accurate values as possible. Every subject was advised to perform their daily activities normally and fall asleep during the hours between 00:00 and 06:00, while keeping the arm sideways, without folding, and as straight and as free as possible. They were also instructed not to perform any intense exercise or sleep during the day. ABPM was carried out during a normal working period for each subject, measuring BP every 15 min during day and every 30 min during night. At least 80% of accurate measurements should have been recorded to ensure valid results. Measurements were interpreted again according to hypertension guidelines [2].

(ii) Laboratory measurements

Laboratory evaluation of certain parameters was conducted in all subjects, including lipid profile, glucose, hemoglobin HbA1c, aminotransferase levels, uric acid and a urinary profile. Regarding the latter, blood urea and creatinine were estimated along with a 24 h urinary sample. Sodium, potassium, urinary creatinine and albumin were analyzed and recorded, and ACR was calculated. Specific instructions for the proper collection of the 24 h urinary sample were given. Participants were instructed to begin collecting their 24 h urine sample after the first morning void and to continue collecting all subsequent urine up to but not including the first void of the following morning. Measurements were interpreted according to the Kidney Disease Improving Global Outcomes guidelines [15,16,17].

(iii) EVAAs

Estimation of EVA was carried out with the new EVAAs, which is based on a Random Forest algorithm that utilizes (a) 24 h Systolic BP, (b) 24 h Diastolic BP, (c) 24 h heart rate, (d) age, (e) sex, (f) BMI, (g) history of diabetes (either yes or no), and (h) eGFR (calculated as MDRD eGFR) to estimate EVA. The parameters were added to the application and the possibility of each patient to have EVA was recorded [18,19].

### 2.3. Statistical Analysis

Qualitative data are presented as numbers (*n*) and respective frequencies (%). For the quantitative data, normality was evaluated using the Shapiro–Wilk test, which is considered to exhibit higher statistical power than the other available tests and is appropriate for small to moderate sample sizes (*n* = 50) [20]. In the case of normally distributed quantitative data the mean and standard deviation (SD) were used for their expression. Otherwise, the median and the interquartile range (IQR) were used. Pearson or Spearman rank correlation coefficients were used to assess the correlation between the different continuous parametric or non-parametric variables, respectively. To assess the correlation between continuous variables and categorical variables, logistic regression was employed, and the statistical significance was evaluated using the likelihood ratio test.

Although no formal a priori sample size calculation was conducted, we performed a *post hoc* power analysis based on the observed correlations. The significance level *α* was chosen at the 0.05 level. To investigate at least moderate correlations (*r* = 0.5), the power of the present study is calculated at 0.97, whereas to investigate at least weak correlations (*r* = 0.3), the power of the study equals 0.57 [20]. Given the small sample size (*n* = 50) and the resulting low observations-per-predictor ratio, we did not perform multivariate regression to avoid overfitting [21,22]. To compare EVAAS for the different groups of patients depending on the variables, smoking, alcohol, hypertension treatment, diabetes, diabetes treatment, dyslipidemia, dyslipidemia treatment and ACR stage, the independent *t*-test was used. The distribution of EVAAS in each of the two groups of the above variables was tested with the Shapiro–Wilk test, and it was found to be normal for each case. The equality of variances (homoscedasticity assumption) was tested with the Levene test. In the case of non-equal variances, Welch’s test was used [23,24,25]. The ANOVA test was used to compare EVAAS for the four different groups of hypertension duration. The distribution of EVAAS in each of the four groups of the above variable was tested with the Shapiro–Wilk test, and it was found to be normal for each case. The homoscedasticity assumption was tested with Levene test, and was found to be valid. All statistical analyses were conducted with SPSS 29.0 (IBM Statistics, Inc., Chicago, IL, USA). Every procedure was in line with the ethical standards of the committee responsible for human experimentation (either institutional or regional) or with the declaration of Helsinki of 1975 (as revised in 1983).

## 3. Results

### 3.1. Descriptive Statistics

Table 1 summarizes the characteristics of the patients included in the present study: 50 patients (48% males) aged 53.7 ± 18.8 participated in this study. Most were non-smokers (66% non-smokers vs. 34% smokers), non-alcohol drinkers (92% vs. 8%), were receiving treatment for hypertension (60 vs. 40%) and were non-diabetic (16% diabetics vs. 84% non-diabetics). The distribution of dyslipidemia was approximately equal (56% no dyslipidemia vs. 44% for previous dyslipidemia), with only 40% of subjects receiving treatment. The mean systolic and diastolic BP measured in the clinic were 140.0 ± 19.3 and 83.0± 13 mmHg, respectively. The mean arterial BP of the study sample, measured in the clinic, was 101.6 mmHg. The mean 24 h ABPM was 123.9 ± 12.9 and 75.5 ± 8.1 mmHg for systolic and diastolic BP, respectively, with a mean 24 h heart rate of 74.9 ± 9.8 bpm. Mean eGFR calculated with MDRD was 79.3 mL/min/1.73 m^2^. ACR was a median of 10.0 mg/g and ACR stage of the sample was mostly normal or high normal (70% normal, 30% moderately increased, 0% abnormal). Measurement of EVAAs for each subject showed a mean value of 0.6 ± 0.2.

### 3.2. Correlation Statistics

Table 2 presents the correlation coefficients for the continuous variables together with the corresponding statistical significance of each correlation. From the Pearson/Spearman correlation coefficient measurements there were four statistically significant correlations with EVAAs (*p* < 0.05). ACR showed a low, but statistically significant correlation with EVAAS (*r* = 0.276, *p* = 0.049). Similarly, serum potassium and serum sodium also showed inverse statistically significant correlations (*r* = −0.29, *p* = 0.038 and *r* = −0.284, *p* = 0.046, respectively) with EVAAS.

Table 3 presents the results of the independent *t*-test or Wilcoxon rank test. From this analysis, again no statistically significant differences were encountered between the EVAAs with any of the variables, though some need to be noted. Most importantly, a normal ACR stage (<30 mg/g) showed a mean difference of 0.090 of lower values of EVAAs than a moderately increased ACR stage (30–300 mg/g) (*p* = 0.075).

## 4. Discussion

In this study, we examined the association between EVA, estimated using EVAAs, and renal damage markers in a hypertensive population. To our knowledge, this is one of the first studies to explore the relationship between EVAAS and albuminuria.

The principal finding was a modest but statistically significant positive correlation between EVAAs and albumin-to-creatinine ratio and may reflect a clinically relevant association within the multifactorial context of vascular aging and renal involvement. Albuminuria is widely recognized as a marker of systemic endothelial dysfunction and microvascular injury and has been consistently associated with arterial stiffness and vascular aging [26]. Experimental and clinical studies suggest that increased albumin excretion reflects structural and functional alterations of the vascular wall, including reduced elastic fiber content and increased vascular calcification, mechanisms that plausibly link albuminuria with arterial stiffening [27,28].

Several population-based studies have demonstrated that even moderately increased albuminuria is associated with increased carotid intima-media thickness and elevated PWV [10,11,29,30]. Our findings extend this concept by suggesting that EVAAS, a non-invasive and easily applicable score, may capture similar pathophysiological information regarding early vascular damage.

Additionally, we observed inverse associations between EVAAs and serum potassium and sodium levels. While the clinical significance of these findings remains uncertain, disturbances in electrolyte homeostasis have been implicated in vascular dysfunction, BP regulation, and arterial stiffness. Previous studies have suggested that both low normal potassium levels and altered sodium balance may contribute to adverse vascular remodeling [31]. These observations, although exploratory, warrant further investigation in larger cohorts.

Taken together, our results support the hypothesis that EVAAs reflects not only vascular aging but also early renal involvement in hypertensive patients. Given its reliance on routinely available clinical data and ABPM, EVAAs could represent a practical tool for identifying patients at increased risk of subclinical target organ damage.

Several limitations should be considered when interpreting our findings. Firstly, the relatively small sample size limits statistical power and precludes multivariable modeling to adjust for potential confounders. Therefore, *p*-values need to be interpreted cautiously given the exploratory nature of these analyses and the number of the associations performed. As no formal multiplicity correction was applied, these findings should be considered hypothesis-generating and warrant verification in larger studies. Secondly, the absence of a normotensive control group restricts our ability to distinguish hypertension-related changes from physiological aging. Thirdly, most participants were receiving antihypertensive treatment, which may have influenced both vascular and renal parameters, introducing residual confounding. Finally, the single-center design and exclusively Caucasian population limit the generalizability of the results.

Given the exploratory pilot design and the limited existing data in this field, these findings should be interpreted as hypothesis-generating and warrant confirmation in larger prospective studies. Specifically, future studies should aim to validate these findings in larger, multicenter cohorts, including untreated hypertensive patients and normotensive controls. Longitudinal designs would be particularly valuable to determine whether EVAAs predicts the progression of renal damage or cardiovascular events over time. In addition, further exploration of the relationship between EVAAs and other forms of target organ damage—such as cardiac remodeling, coronary artery disease, and heart failure—may clarify its role as a comprehensive cardiovascular risk assessment tool.

## 5. Conclusions

In conclusion, EVAAs was associated with albuminuria and electrolyte parameters indicative of early renal involvement in a hypertensive population. These findings suggest that EVAAs may serve as a simple, non-invasive marker of early vascular and renal target organ damage. Larger prospective studies are required to confirm its clinical utility and prognostic value.

## Figures and Tables

**Table 1:** Characteristics of the patients included in the present study.

Sociodemographic		
Sex, *n* (%)	Male	24 (48.0)
	Female	26 (52.0)
Age (years), mean ± SD	53.7 ± 18.8	
Weight (kg), mean ± SD	82.7 ± 19.6	
Height (cm), mean ± SD	170.6 ± 9.8	
Waist (cm), mean ± SD	99.1 ± 17.2	
Hip (cm), mean ± SD	109.1 ± 17.3	
BMI, mean ± SD	28.3 ± 5.5	
Smoking, *n* (%)	No	33 (66.0)
	Yes	17 (34.0)
Alcohol use, *n* (%)	No	46 (92.0)
	Yes	4 (8.0)
Hypertension duration (months), *n* (%)	<1	13 (26.0)
	1–24	16 (32.0)
	24–60	11 (22.0)
	>60	10 (20.0)
Hypertension treatment, *n* (%)	No	20 (40.0)
	Yes	30 (60.0)
Diabetes treatment, *n* (%)	No	44 (88.0)
	Yes	6 (12.0)
Dyslipidemia treatment, *n* (%)	No	30 (60.0)
	Yes	20 (40.0)
BP (mean ± SD)		
Mean systolic BP measured at the clinic (mmHg)	140.0 ± 19.3	
Mean diastolic BP measured at the clinic (mmHg)	83.0 ± 13	
Mean systolic BP measured for 24 h (mmHg)	123.9 ± 12.9	
Mean diastolic BP measured for 24 h (mmHg)	75.5 ± 8.1	
Mean heart rate measured for 24 h (bpm)	74.9 ± 9.8	
Mean systolic BP measured for 24 h—day hours (mmHg)	126.9 ± 13.6	
Mean diastolic BP measured for 24 h—day hours (mmHg)	78.2 ± 8.6	
Mean heart rate measured for 24 h—day hours (bpm)	77.7 ± 10.8	
Mean systolic BP measured for 24 h—night hours (mmHg)	114.1 ± 11.9	
Mean diastolic BP measured for 24 h—night hours (mmHg)	69.9 ± 8.6	
Mean heart rate measured for 24 h—night hours (bpm)	68.8 ± 9.9	
Blood Test Results (mean ± SD)		
HbA1c (%)	5.6 ± 3	
Cholesterol (mg/dL)	194.1 ± 48.1	
Triglycerides (mg/dL)	132 ± 18	
HDL-C (mg/dL)	57.5 ± 13.8	
LDL-C (mg/dL)	99.5 ± 8	
Creatinine (mg/dL)	0.9 ± 0.2	
eGFR-MDRD (mL/min/1.73 m^2^)	79.3 ± 9	
Potassium (mEq/L)	4.3 ± 0.4	
Sodium (mEq/L)	139.8 ± 2.3	
Uric acid (mg/dL)	6.0 ± 1.6	
EVAAs (mean ± SD)		
EVAAs, mean ± SD	0.6 ± 0.2	
Urine Test Results (mean ± SD)		
Creatinine (mg/24 h)	1 457.0 ± 400	
Albumin (mg/24 h)	10.3 ± 5	
Potassium (mmol/24 h)	54.3 ± 18	
Sodium (mEq/24 h)	145.5 ± 49.1	
ACR (mg/g)	10.0 ± 8	
ACR stage, *n* (%)	Normal	35 (70.0)
	Moderately increased	15 (30.0)
	Abnormal	0 (0.0)

**Table 2:** Correlation coefficients for the correlation between blood and urine test results, continuous variables, and EVAAs.

Continuous Variable	Pearson/Spearman, *r* (-)	*p*-Value (-) *, 95%CI
Serum Potassium	−0.290	0.038 (−0.530, −0.018)
Serum Sodium	−0.284	0.046 (−0.521, −0.006)
Urine Test Results		
Creatinine	0.120	0.408 (−0.136, 0.384)
Albumin	−0.152	0.292 (−0.432, 0.129)
Potassium	0.009	0.949 (−0.271, 0.271)
Sodium	0.220	0.132 (−0.066, 0.466)
ACR	0.276	0.049 (0.019, 0.520)

* Statistical significance at 0.05 level.

**Table 3:** Results of the independent *t*-test for EVAAs between normal and high normal ACR stage.

Variable	Comparison	*t*-Test/Welch’s *p*-Value	Mean Difference (95%CI)
ACR stage	Normal– Moderately increased	0.075	0.090 (−0.009, 0.189)

## Data Availability

The data presented in this study are available on request from the corresponding author. The data are not publicly available due to privacy.

## Abbreviations

The following abbreviations are used in this manuscript:

WHO	World Health Organization
BP	Blood Pressure
PWV	Pulse Wave Velocity
EVAAS	Early Vascular Aging Ambulatory score
CKD	Chronic kidney disease
ABPM	Ambulatory Blood Pressure Monitoring
BMI	Body Mass Index
eGFR	estimated Glomerular Filtration Rate
ACR	Albumin-to-creatinine ratio
EVA	Early Vascular Aging
GFR	Glomerular filtration rate
